# Single vs. Combined Therapeutic Approaches in Rats With Chronic Spinal Cord Injury

**DOI:** 10.3389/fneur.2020.00136

**Published:** 2020-03-10

**Authors:** Vinnitsa Buzoianu-Anguiano, Jared Rivera-Osorio, Sandra Orozco-Suárez, Angélica Vega-García, Elisa García-Vences, Stephanie Sánchez-Torres, Ismael Jiménez-Estrada, Gabriel Guizar-Sahagún, Jose Mondragon-Caso, Francisca Fernández-Valverde, Ignacio Madrazo, Israel Grijalva

**Affiliations:** ^1^Hospital de Especialidades CMN Siglo XXI IMSS, Unidad de Investigación Médica en Enfermedades Neurologicas, Mexico City, Mexico; ^2^Centro de Investigación en Ciencias de la Salud, Universidad Anahuac México Campus Norte, Mexico City, Mexico; ^3^Departamento de Fisiología, Biofísica y Neurociencias, CINVESTAV, IPN, Mexico City, Mexico; ^4^Departamento de Cirugía Experimental, Proyecto Camina AC, Mexico City, Mexico; ^5^Laboratorio de Patología Experimental, Instituto Nacional de Neurología y Neurocirugía, Mexico City, Mexico

**Keywords:** axonal regeneration, bone marrow stromal cell transplant, pre-degeneration peripheral nerve transplant, BBB modified score, kinematic analysis, functional recovery

## Abstract

The regenerative capability of the central nervous system is limited after traumatic spinal cord injury (SCI) due to intrinsic and extrinsic factors that inhibit spinal cord regeneration, resulting in deficient functional recovery. It has been shown that strategies, such as pre-degenerated peripheral nerve (PPN) grafts or the use of bone marrow stromal cells (BMSCs) or exogenous molecules, such as chondroitinase ABC (ChABC) promote axonal growth and remyelination, resulting in an improvement in locomotor function. These treatments have been primarily assessed in acute injury models. The aim of the present study is to evaluate the ability of several single and combined treatments in order to modify the course of chronic complete SCI in rats. A complete cord transection was performed at the T9 level. One month later, animals were divided into five groups: original injury only (control group), and original injury plus spinal cord re-transection to create a gap to accommodate BMSCs, PPN, PPN + BMSCs, and PPN + BMSCs + ChABC. In comparison with control and single-treatment groups (PPN and BMSCs), combined treatment groups (PPN + BMSCs and PPN + BMSCs + ChABC) showed significative axonal regrowth, as revealed by an increase in GAP-43 and MAP-1B expression in axonal fibers, which correlated with an improvement in locomotor function. In conclusion, the combined therapies tested here improve locomotor function by enhancing axonal regeneration in rats with chronic SCI. Further studies are warranted to refine this promising line of research for clinical purposes.

## Introduction

Traumatic Spinal Cord Injury (SCI) results in partial or complete alterations of motor, sensation, and autonomic functions. Despite the advances in biomedical research for acute SCI, there is no effective treatment for axonal regeneration and functional restoration at chronic stages.

After SCI, efficient cord regeneration is mainly limited by a local inhibitory environment due in part to a biochemical barrier formed by molecules like myelin-associated proteins (1, 2) as well as proteins constitutive of the extracellular matrix, markedly chondroitin sulfates (3, 4). In addition, a physical barrier (fibrogial scar) formed by microglia, oligodendrocytes, meningeal cells, and reactive astrocytes also inhibits axonal regrowth (5, 6).

Some strategies for promoting axonal regeneration and functional restoration in spinal cord injury models are relevant to the present study, especially those based on bone marrow stromal cell (BMSC) transplantation (7), fresh and pre-degenerated peripheral nerve (PPN) transplantation (8), and chondroitinase ABC (9, 10), all of which promote partial neuroprotective and regenerative effects after early SCI.

If used independently, the benefits of the above-mentioned strategies seem insufficient to be translated to patients, but several experimental studies have shown that combined therapies may produce a greater benefit (11–14). In fact, recently, we reported on the additive morphofunctional effect of combining PPN and BMSCs in a model of spinal cord transection (15). This justifies continuous testing for this kind of strategy.

To continue along this promising research line, the current study is designed to test the hypothesis that the combination of ChABC with PPN and BMSC transplant has a synergic effect on axonal regrowth and functional recovery in a model of complete chronic spinal cord transection. Our objective is to evaluate the reparative ability of several single and combined treatments targeting some barriers for spinal cord regeneration in rats with sequelae of severe and delayed SCI.

## Methods

### Experimental Design

The Ethics Committee in Research of the Hospital de Especialidades, Centro Médico Siglo XXI, Instituto Mexicano del Seguro Social approved this study. Experiments were performed according to the official Mexican norm NOM-062. A total of 114 syngeneic Fisher 344 rats, 8–10 weeks old, and 200–220 g body weight were used. For SCI and transplants, 58 female rats were randomly divided into five groups. Twenty-eight male rats were used as sciatic nerve donors, and 28 male rats were used for BMSC procurement. Male donors were used for PPN transplants; as the nerve itself is larger than female nerves, we only used a single nerve to fill in the gap. After all surgical procedures, animals received oral enorfloxacin 10 mg/kg and ibuprofen 15 mg/kg once a day for 8 days.

### Surgical Procedures and Transplant Preparation

#### Spinal Cord Injury

For cord injury, rats were anesthetized with a mixture of ketamine (70 mg/kg) and xylazine (10 mg/kg) given IM. A laminectomy was performed at T9, and subsequently, the dorsal portion of the dural sac was longitudinally sectioned and the spinal cord was completely transected in the transverse plane with microsurgery scissors. Complete transection was verified using a microsurgery hook to check for the absence of residual tissue. Finally, the skin wound was sutured, and the animal was returned to its home cage.

#### Peripheral Nerve Preparation for Transplantation

Twenty-one days before transplant, donor rats were anesthetized and subjected to complete transversal section of the sciatic nerve at the cephalic portion of the thigh; the free end of the caudal stump of the sectioned nerve was sutured with 7-0 nylon to prevent spontaneous nerve regeneration. On transplant day, rats were anesthetized to remove an ~2 cm long segment of the sciatic nerve distal to the section. The nerve was then placed in ice-cold isotonic saline solution until transplantation.

#### Isolation and Culture of Bone Marrow Stromal Cells

BMSCs were obtained from the femurs and tibias aseptically harvested from donor rats euthanized with an overdose of sodic pentobarbital. Bone marrow was obtained by rinsing out each harvested bone with growing medium (Dulbecco's Modified Eagle D-MEM® GIBCO) using a 3-ml syringe; the released marrow was then centrifuged for 7 min at 1,300 rpm. The BMSCs were separated by centrifugation at 2,000 rpm at 24°C for 45 min with Ficol gradient (SIGMA) (3 ml). The cells obtained were seeded in a 75-cm^2^ culture flask with 10 ml of D-MEM, 20% bovine serum (FSB, GIBCO), 2 ml L-glutamine (GIBCO), 2 ml antibiotic-antimycotic (GIBCO), and 2 ml of non-essential amino acids (GIBCO). Cells were then placed in a water-jacketed incubator at 37°C with 5% CO_2_ until a monolayer of fibroblasts was formed. Afterward, a layer of BMSC on top of the fibroblasts was seeded, and cultures were kept for four passes until they became mature.

#### Transplantation

Four weeks after spinal cord transection, rats were randomly assigned to one of the five experimental groups. In Group 1 (control, *n* = 14), the surgical wound was reopened, and the dural sac was exposed without removing the scar. In Group 2 (*n* = 14), the dural sac was reopened in order to carefully remove the scar, leaving an ~6-mm gap. Then, two injections of 5 μl each of DMEM medium (GIBCO) containing 3 × 10^4^ BMSCs were injected parasagittally on each stump of the spinal cord with a 2 mm depth at the edge of both the rostral and caudal stumps. In Group 3 (*n* = 14), the spinal cord scar was removed as described for Group 2, and then, three to five PPN segments, each 6 mm long, were longitudinally implanted in the spinal cord gap. The implants were affixed with fibrin glue (BAXTER). In Group 4 (*n* = 14), the treatments described in Groups 2 and 3 were combined. Finally, in Group 5, the treatment described in Group 2 was used, with two BMSC injections administered in each stump, but adding 6 μl of ChABC (2 units/ml Seikagaku 100332; Associates of Cape Cod) to each injection (injecting a total volume of 11 μl) and with PPN implanted in the cord gap as described in Group 3.

### Assessment of Locomotor Performance

#### Open Field Test

Hind limb locomotion was assessed by the modified BBB score for complete transaction: 22 points in four levels were evaluated, focusing on rhythmicity, movement alternation with and without body weight, and plantar support (16). Animals were evaluated 24 h after injury and weekly for the following 12 weeks. Observers were blind to experimental groups, and the rats were not placed on a treadmill for the open field test.

#### Kinematic Analysis

Kinematic registration of gait was performed after 3 months of treatment. First, hindlimbs were marked with a non-toxic marker (Sharpie®) on the iliac crest, hip, knee, ankle, and fifth metatarsal phalangeal joints. Next, each animal was introduced separately into an acrylic tunnel (60 × 5 × 5 cm) marked every 5 cm. The animal was then recorded with a commercial digital video camera. Four consecutive steps (the first step was not considered in order to exclude the initial phase of the gait) were analyzed. With the help of computer software (Total Video Converter), digital photographs were obtained from each recording frame (30 frames/s). The Cartesian coordinates of each joint were determined from each photograph by using ImageJ software (Version 1.30, NIH). The registered coordinates were converted from pixels to centimeters based on the reference marks (5 cm) placed in the tunnel.

The values in centimeters were introduced into a software platform designed by CINVESTAV-IPN (17), which associates joints defined by Cartesian coordinates, drawing all the resulting lines of the movement executed by the animal's limbs during gait and the movement sequence of the extremities during walking.

### Procedures for Morphological Assessments

#### Immunofluorescence

Twelve weeks after treatment, animals were euthanized with sodic pentobarbital IP 40 mg/kg and, immediately after, were intracardially perfused with 0.9% NaCl followed by 4% paraformaldehyde. A 2-cm-long segment of cord centered at the site of injury (Supplementary Figure 1) was removed. Tissues were placed in PBS with 30% sucrose for 24 h. Next, 20-μm-thick serial sagittal sections were obtained with a LEICA cryostat. Sections were washed in PBS and were then blocked with normal bovine serum (Vector Lab) (1:200 in PBS) for 30 min. They were then incubated in anti-microtubule-associated protein 1B (MAP1-B, C-20, goat polyclonal IgG, Catalog no. sc-8971; Santa Cruz Biotech), anti-growth associated protein 43 (GAP-43, H-100, rabbit polyclonal IgG, Catalog no. sc-1779), anti-glial fibrillary acidic protein (GFAP, H-50, rabbit polyclonal IgG, Catalog no. sc-65343), and anti-NGFR p75 (mouse monoclonal IgG, Catalog no. sc-71692) for 48 h at 4°C. Samples were washed with PBS and incubated with the secondary antibody (Alexa 488 Anti-rabbit or Anti-goat, Molecular Probes Invitrogen, Catalog no. A 11008) for 2 h; finally, they were again washed with PBS, contrasted with Hoechst (dilution of 5 μl/5 ml PBS; Molecular Probes, Life Technologies) for 3 min, and covered with Vectashield (Vector Lab) and a cover glass. Images were acquired using a Nikon Ti Eclipse inverted confocal microscope equipped with an A1 imaging system, controlled with the proprietary software NIS Elements v.4.5.0. Imaging was performed using either a 20 × (Dry, NA 0.8) or 60 × (Oil immersion, NA 1.4) objective lens. Dyes were excited in sequential mode using the built-in laser lines 403 nm (Hoechst), 488 nm (Alexa 488), and 563 nm (Alexa 546). The corresponding fluorescence was read in the following ranges: 425–475 nm (Hoechst 33342), 500–550 nm (Alexa fluor 488), and 570–620 nm (Alexa fluor 546) using the manufacturer-provided filter sets. The images were acquired and analyzed using NIS Elements V.4.50. From each section, six images were acquired, comprising the epicenter and the zones rostral and caudal to the site of injury. Using FIJI-ImageJ software, the intensity of green-channel pixels, corresponding to the Alexa 488 per area, was quantified. The calibration of the intensity by area was conducted via a spatial scale derived by using the intensity values contained within the Images bitmap (image given by the Software) to establish the basal intensity values of each image. The density is the same as the intensity per area, which was determined in relation to the control groups and expressed as pixels/mm^2^; the counting area was adjusted to 1 mm^2^.

#### Tracers to Assess Axonal Regrowth

Twelve weeks after treatment, one anterograde neurotracer in the brain and one in the spinal cord were injected into two animals from each group. Anesthetized animals underwent stereotactic surgery by drilling the skull according to the following coordinates: (−1.2 mm) anteroposterior, lateral right (−2 mm), and lateral left (+2 mm) with respect to Bregma. The tracer was injected through a micro-injector (Kd Scientific 310-plus), connecting a Hamilton syringe adapted with a microdialysis tube. The tip of the syringe was placed on the brain surface and introduced 2 mm in the drilled canals. Two microliters of mini-ruby dextran tetramethylrhodamine (Molecular Probes Life Technologies, Catalog no. D3312) were injected, at a rate of 0.5 ml/min on each side, and the skin was sutured. Afterward, the spinal cord was exposed through a laminectomy performed at T12 to inject mini-emerald dextran fluorescein trace (Molecular Probes Life Technologies, D7178). Using a stereotactic approach to the spine, a microinjection was performed in the spinal cord, close to the central artery, 1 mm from the surface; a total volume of 1 μl of the solution was delivered at a rate of 0.01 ml/min. The skin was sutured, and the animal was returned to its home cage. After 3 weeks, animals were intracardially perfused with 0.9% NaCl followed by 4% paraformaldehyde.

Twenty-micron longitudinal floating sections were obtained in a cryostat. After collection, sections were contrasted with Hoechst (dilution of 5/5 ml of PBS; Molecular Probes) and mounted with Vectashield. The tissue was analyzed with a confocal microscope to identify the stained axons with neurotracers.

### Statistical Analysis

The one-way ANOVA on ranks (Kruskall-Wallis tests) and Mann-Whitney *U* tests were conducted when the data distribution required non-parametric testing. Locomotor function (BBB) data were analyzed using the repeated measures ANOVA test. Kinematics data were analyzed using a two-way ANOVA test followed by Tukey's test. Statistical analyses were performed using Graph Pad Prism V6.0. A significance level of *p* < 0.05 was set for all analyses.

## Results

### Morphological Outcome

#### Phenotypification of BMSCs

**Figure 1:**
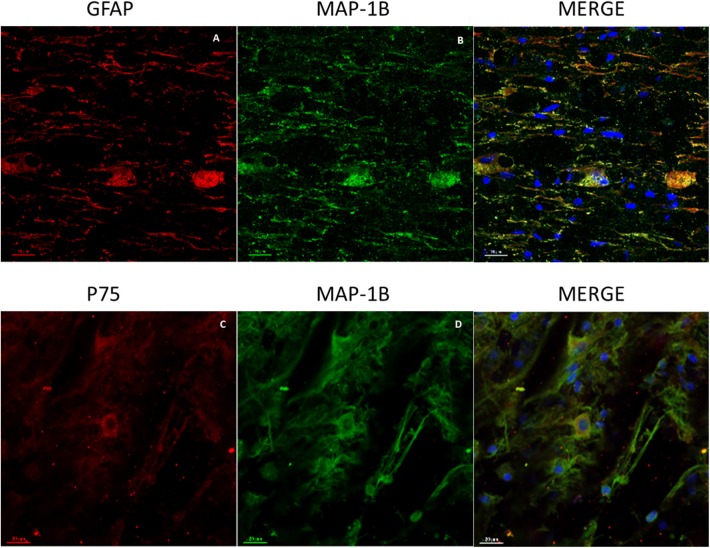
Representative images of GFAP, MAP-1B, and P75 expression in BMSCs at the spinal cord. **(A)** Immunoreactive cells for GFAP (red). **(B)** Immunoreactive cells for MAP-1B (green). **(C)** Immunoreactive cells for P75 with star-like morphology (red). **(D)** Immunoreactive cells for MAP-1B with long extensions (green). Nuclei contrasted with Hoechst (blue). Scale bars **(A–D)** = 50 μm.

Cells were characterized by the immunophenotyping technique (flow cytometry), showing that 66.5% of the population was positive to Cd90 2.85% was double-positive for Cd105 and Cd13, and 37.4% was positive for Cd13 demonstrating that the population used for transplants were adult mesenchymal stem cells (Supplementary Figure 2A). In the spinal cord tissue, all groups treated with BMSCs, regardless of whether they had received single or combined treatment, showed Cd90- and Cd105-positive cells in the spinal cord, grouped into colonies (Supplementary Figures 2B,C). In addition, these cells showed immunoreactivity for GFAP, MAP-1B, and P75 (Figures 1A–C) and displayed a glial star-shaped morphology with long ramifications (Figure 1D). These cells were absent in rats that did not receive BMSCs.

#### Axonal Regeneration

**Figure 2:**
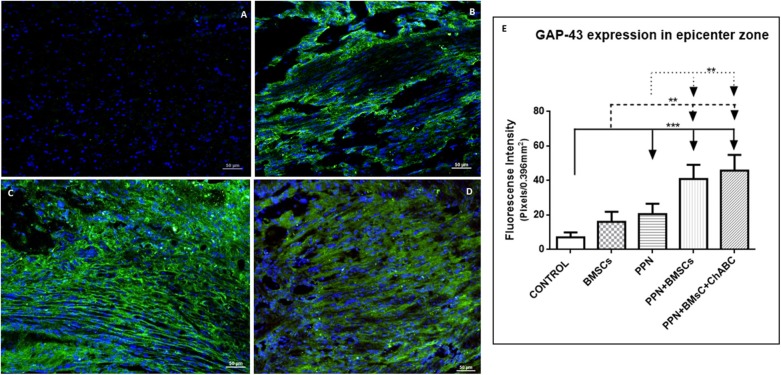
Expression of GAP-43 at the epicenter. **(A–D)** Show representative images of immunoreactive fibers for GAP-43 (green) of groups control **(A)**, PPN **(B)**, PPN + BMSCs **(C)**, and PPN + BMSCs + ChABC **(D)**. Note that fibers in the PPN group seem disordered, scarcer, and thinner compared to combined groups. Scale bar **(A–C)** = 50 μm. Plots in **(E)** show quantification of fluorescein density at the epicenter zone. Data are expressed as the mean ± S.D. (*n* = 6). Statistical analysis: Kruskal Wallis followed by *U*-Mann Witney. ***p < 0.0001; **p < 0.05.

The qualitative exploration (analysis and observations) of GAP-43 expression revealed that the apparent amount of immunoreactive fibers in the control group (Supplementary Figures 3A,F) was less than that observed in groups with single treatment (Supplementary Figures 3B,C,G,H). In addition, in the control groups, such fibers appear disorganized. Qualitatively comparing combined (Supplementary Figures 3D,E,I,J) against single treatment groups, it seems that the immunoreactive fibers are more abundant, of larger caliber, and better organized after combined treatments (Figures 2A–D).

Quantitative analysis for GAP-43 confirms the above-mentioned observations. At the epicenter (Figure 2E) and in areas rostral and caudal to it (Supplementary Figures 4A,B), the fluorescein density of all treated groups was significantly higher than in the control group (*p* < 0.001). Density was also significantly higher for the combined treatment groups when compared to those receiving single treatments (*p* < 0.05).

**Figure 3:**
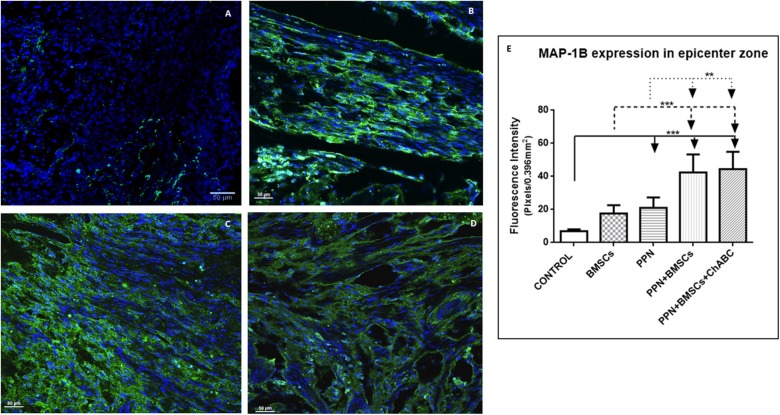
Expression of MAP-1B at the epicenter. **(A–D)** Show representative images of immunoreactive fibers for MAP-1B (green) of groups control **(A)**, PPN **(B)**, PPN + BMSCs **(C)**, and PPN + BMSCs + ChABC **(D)**. Note that fibers in the PPN group seem disordered, scarcer, and thinner compared to combined groups. Scale bar **(A–C)** = 50 μm. Plots in **(E)** show quantification of fluorescein density at the epicenter zone. Data are expressed as the mean ± S.D. (*n* = 6). Statistical analysis: Kruskal Wallis followed by *U*-Mann Witney. ***p < 0.0001; **p < 0.05.

For MAP-1B expression, the same qualitative and quantitative trends were observed: the greatest quantity of positive immunoreactive fibers and the best organization of these was observed in the combined-treatment groups, followed by the single-treatment groups, and finally in the control group (Figures 3A–D and Supplementary Figure 5). Quantitative analysis revealed that the fluorescein density of all treated groups was significantly higher than in the control group (*p* < 0.001) and was significantly higher in combined treatments than in single treatments (*p* < 0.05) (Figure 3E and Supplementary Figures 4C,D).

**Figure 4:**
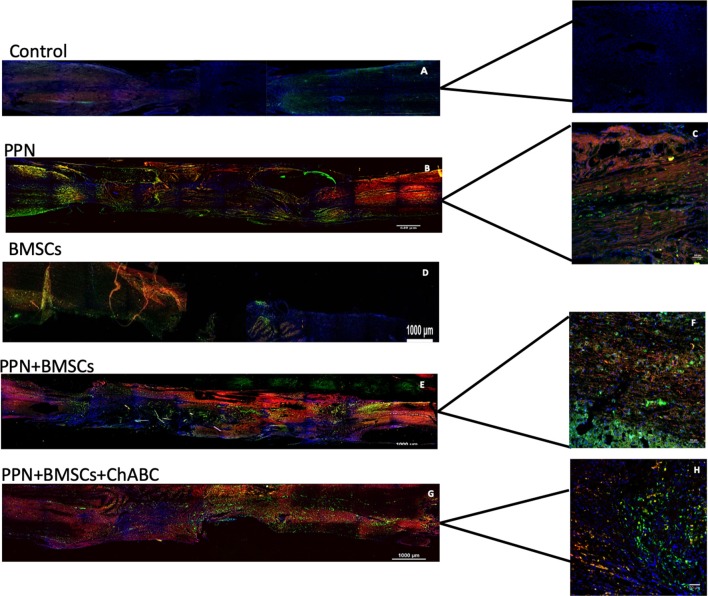
Representative images of axonal regeneration. In **(A,B,D,G)**, descending fibers (red, mini-ruby dextran, tetramethylrhodamine) and ascending fibers (green, emerald fluoro dextran fluorescein) are shown in the rostral zone (left), epicenter (central zone), and caudal zone (right) of the spinal cord. In **(C,F,H)** positive fibers from each tracer are observed in the transplant zone (central zone). Nuclei dyed in blue with Hoechst. Scale for **A,B,D,F** = 1,000 μm and for **C,E,G** = 50 μm.

**Figure 5:**
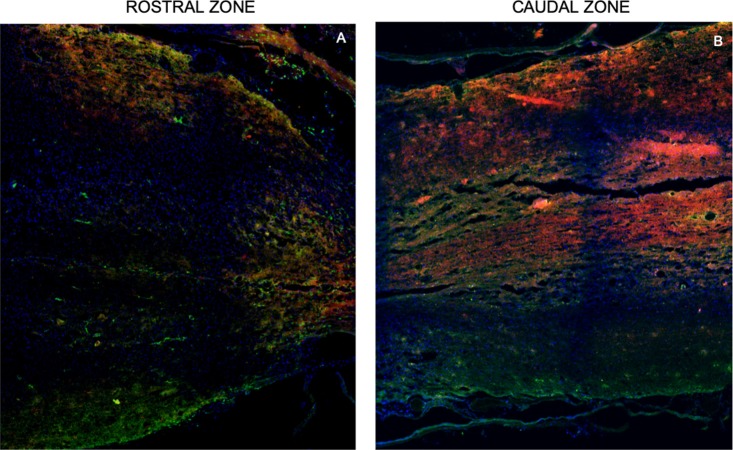
Representative tracing images of the spinal cord. Rostral zone **(A)** and caudal zone **(B)** of the spinal cord show ascending fibers (red, mini-ruby dextran, tetramethylrhodamine) and descending fibers (green, emerald fluoro dextran fluorescein). These inmunofluorescence are representative of the staining of the neurotracers. Nuclei dyed in blue with Hoechst. Scale = 1,000 μm.

Neurotracers in specimens of rats containing PPN as a graft, both with single and combined treatment, showed, at the epicenter as well as rostral and caudal to it, fibers marked with ruby red (Fluoro mini-ruby dextran, tetramethylrhodamine) belonging to the motor area and green emerald fibers (Fluoro emerald dextran fluorescein) coming from the lumbar area. Positive fibers for both tracers appear better organized, thicker, and increased in combined treatment compared to single treatment (Figures 4, 5).

#### Histological Appearance of Host-PPN Apposition

At the end of the experiment, groups with pre-degenerate peripheral nerve transplantation showed a good apposition between nerve graft and spared spinal cord, with the presence of micro-cysts in both rostral and caudal interface zones; the opposite features were seen in the control group (Supplementary Figure 6).

### Locomotor Function Outcome

**Figure 6:**
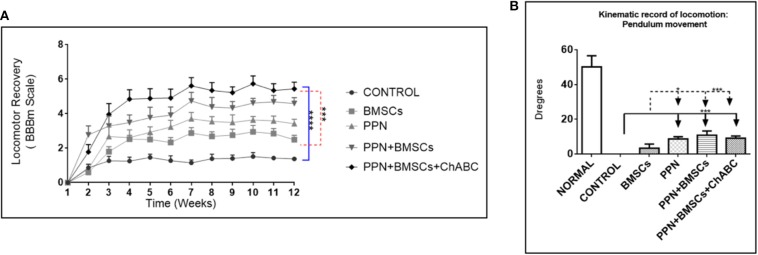
Effect of therapeutic approaches on locomotor function. **(A)** Shows the modified BBB scores obtained during 12 weeks after treatment. Statistical analysis: repeated-measures ANOVA followed by Tukey test. ****p < 0.0001; ***p < 0.001. **(B)** Shows kinematic recording of pendular movement. Statistical analysis: two-way ANOVA followed by Tukey test. ***p < 0.0001; *p < 0.05. Data are expressed as the mean ± S.E.M. (*n* = 12).

The modified BBB scale showed that all treated groups scored significantly higher than the control group (*p* < 0.0001; Figure 6A). Combined treatment groups showed a significantly higher score than the single treatment group (*p* < 0.001). Remarkably, from the 8th week onwards, combined treatment groups showed frequent rhythmic movements in both hindlimbs.

**Figure 7:**
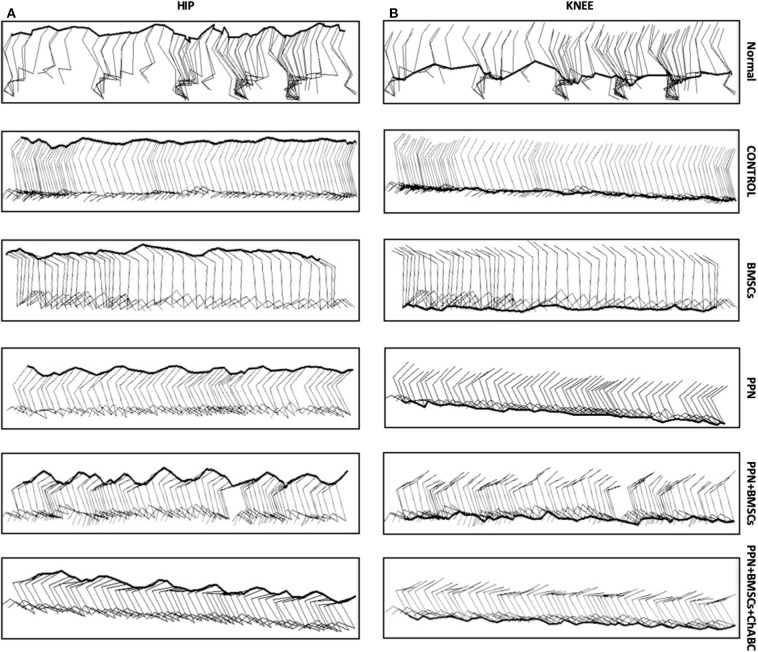
Representative sagittal stick diagrams of kinematic gait recording. **(A)** Hip; **(B)** knee. Diagrams in intact rats show the movement of the hips and knees is in waveforms. In the control groups, diagrams show no movements; only flat lines can be seen. In the group treated with BMSC, waves are inconsistent and have small amplitude. With PPN treatment, there is an increase in the formation of waves in both joints. Noticeably, in the combined treatment (PPN + BMSCs and PPN + BMSCs + ChABC) groups, the waves show greater amplitude and are constant in both joints. The images of Normal, Control, PPN, PPN + BMSCs, and PPN + BMSCs + ChABC represent the right leg, and only the group for BMSCs represents the left leg.

The kinematic analysis revealed a significant increase in the amplitude of hindlimb movements in groups treated with PPN grafts alone and with combined treatments in comparison to the control and BMSC groups (*p* < 0.0001) (Figure 6B). The increase in the amplitude of movements was observed in the gait pendulum movement (stride) in combined groups, which was greater than in the single treatment and control groups (Supplementary Figure 7). This increase in the amplitude of movements was shown in both hips and knees (Figures 7A,B).

## Discussion

This study was designed to explore the potential of combined therapeutic approaches for promoting neural regeneration after complete chronic spinal cord transection, as assessed by analysis of the expression of molecules associated with axonal growth, neural tracing, and locomotor performance.

### Rationale for Experimental Design

#### Choice of Therapeutic Procedures

The choice of therapeutic procedures tested here was based on previous reports claiming their ability to support spinal cord regeneration, each through different mechanisms.

BMSCs support axonal regrowth in both acute (18, 19) and chronic (20) cord transection. BMSCs are capable of building a niche inside the host from which diverse growth factors and molecules for axonal guidance and extension are secreted, promoting an enabling environment for axonal regrowth (21–23). In addition, BMSCs promote oligodendrogenesis (24).

The tubular structure and cells (mainly Schwann cells and macrophages) present in implants of PPN work as scaffolding and promote a permissive microenvironment for axonal regrowth in both acute and chronic spinal cord transection (25–27). Regenerated axons are able to cross both rostral and caudal graft-host interfaces (8, 15, 28, 29).

ChABC digests the glycosaminoglycan chains of chondroitin sulfate and restores plasticity to the adult spinal cord and brain, making axonal regrowth possible (9, 30). The power of this enzyme to digest the chondroitin sulfate proteoglycans that are associated with a glial scar has been exploited to facilitate the extent of axons between grafts and neighboring spinal cord (31).

#### Injury Model and Assessments

In order to study axonal regeneration in SCI, one must work with a partial or complete cord transection. Because of the enormous variability observed in the clinical setting, the experimental model used here appears to be a suitable alternative for studying the influence of combined therapeutic approaches, as it maintains uniformity in both the severity of injury and the time elapsed before starting treatment.

The most appropriate histological methods for evaluating axonal regeneration involve the use of neurotracers, as well as the expression of molecules directly or indirectly associated with both guiding and assembling the regenerating axons, such as GAP-43 and MAP-1B (32–34).

Although the standard BBB scale is by far the most commonly used test for motor assessment in experimental rodent SCI, here we choose a modified version of BBB design for complete transection because of its greater sensitivity to detecting movements originating distally to the site of injury (16, 35). In addition, the kinematic analysis of movement provides a valuable complementary assessment, as it is objective and accurate (36).

#### Injury Chronicity

Performing a therapeutic intervention in chronic stages of SCI, as in this study, represents a greater challenge for successfully promoting cord regeneration compared to earlier therapeutic interventions.

A lot of experimental strategies for cord repair at acute stages have been tested, including cell and tissue transplants, exogenous substances capable of neutralizing neurite outgrowth inhibitors like myelin-associated proteins and physical barriers, growth factors, biomaterials, and drugs (37, 38). Many of these treatments promote axonal regrowth and improve locomotor function, with different extents of efficiency. Similar treatment strategies have been tested in a limited number of studies in subjects with injury at chronic stages, usually with poorer results (9, 20, 27).

### Single vs. Combined Treatments

It was shown here that by using combined treatments, axonal regeneration, as well as locomotor function recovery, increases in comparison to single treatments. These results confirm our hypothesis and underline the suitability of simultaneously targeting different barriers for spinal cord regeneration to obtain better results.

Our results are consistent with previous studies reporting that, despite the positive structural and functional results obtained with single treatments, their combination has an additive effect, improving individual positive effects (39, 40).

Some successful combinations that promoted greater axonal regeneration than single treatments include treatments combining PPN + FGFα (41), PPN + ChABC (31), PPN + ChABC and PPN + BDNF (29), BMSCs + granulocyte-colony stimulating factor (42), ChABC + NT3 (13), ChABC + rehabilitation (9), and PPN + BMSC (15).

In most studies where the effect of combined treatment for SCI has been evaluated, two procedures have been used. In the few studies where three procedures were combined, the result was better in comparison to single or dual interventions (13, 43), which supports the concept that certain combinations may have an additional additive effect, as observed in this study.

### Morpho-Functional Outcome

As expected, the highest axonal regeneration corresponds with the greatest functional recovery, as observed in animals that received combined treatments.

Our observation from cytometry that PPN bridges placed in the site of injury both increase the number and support the axial orientation of regenerated axons emerging from both stumps agrees with previous studies reporting similar results from using cord hemisections treated in the acute stage, with PPN combined with other interventions (29, 44). Functionally, treatment using only PPN promotes mild locomotor function recovery in both acute (45, 46) and chronic stages (15). Similarly, the mild motor recovery observed here after single treatment with BMSCs in the chronic stage of injury agrees with the functional outcome after transplanting only BMSCs in the acute phase of injury (11, 42, 47).

As we have shown here by combining PPN + BMSCs and PPN + BMSCs + ChABC, others have previously reported that the augmentation of locomotor function obtained through single treatments is potentiated by combined treatments. An example of such benefit with treatments given early after injury are the combinations of PPN + FGFα (44), PPN + ChABC (45), and ChABC + NT-3 (13). Examples of studies where the benefit was obtained after administering treatment in chronic stages of the lesion are the combinations of PPN + BMSCs (15), BMSCs + Chitosan (11), BMSCs + G-CSF (42), and cADMSCs + ChABC (48).

Although we did not determine the precise mechanisms behind the axonal regeneration and functional recovery observed with combined treatments, it is reasonable to speculate that functional reinnervation may be established after the PPN promotes long-distance axonal regeneration, while, in the interphase areas, BMSCs release molecules capable of enhancing axonal outgrowth and providing guidance for synapse formation on appropriate targets (49). Finally, the administration of ChABC did not produce the expected beneficial additive effect as part of a combined treatment, suggesting that the administration strategy was insufficient.

### Limitations and Future Prospects

While we were able to demonstrate an initial impression of the potential for our approach to regenerate the spinal cord in the late stage of injury, a limitation of the present study is the small sample size, which only allows preliminary conclusions to be drawn. Nonetheless, we believe that our results provide a basis for future studies aimed at improving this encouraging therapeutic approach and fully developing its applicability.

To gain an initial perspective on the effects of the approach tested here, histology and locomotor evaluations were performed. However, more in-depth mechanistic studies are warranted for a better understanding of how combined treatments might modify key areas within the microenvironment of the injury, including myelination and other molecular events of these therapies.

### Conclusion

Using a clinically relevant model of chronic SCI in rats, we have investigated the morpho-functional effects of single and combined therapeutic procedures targeting different barriers for spinal cord regeneration.

In the long run, PPN and BMSCs given as single treatments discretely enhance axonal regeneration and locomotor function, while, by combining treatments (PPN + BMSCs and PPN + BMSCs + ChABC), a significant enhancement of axonal regeneration and a modest recovery of locomotion was observed. These findings suggest that functional spinal cord regeneration can be effectively induced in the late stages of injury.

The encouraging results reported here merit further pre-clinical studies designed to delve into this promising line of research. In addition, discernment of the precise mechanisms underlying these benefits deserves further attention.

## Data Availability Statement

All datasets generated for this study are included in the article/Supplementary Material.

## Ethics Statement

The animal study was reviewed and approved by the Ethics Committee in Research of the Hospital de Especialidades, Centro Médico Siglo XXI, Instituto Mexicano del Seguro Social.

## Author Contributions

VB-A: conception and design, provision of study material, collection and/or assembly of data, data analysis and interpretation, and manuscript writing. JR-O, EG-V, SS-T, and IJ-E: collection and/or assembly of data and data analysis and interpretation. SO-S: conception and design, provision of study material, data analysis and interpretation, and final approval of the manuscript. AV-G: collection and/or assembly of data. GG-S: data analysis and interpretation and manuscript writing. JM-C and IM: manuscript writing. FF-V: collection and/or assembly of data. IG: conception and design, provision of study material, financial support, administrative support, and final approval of the manuscript.

### Conflict of Interest

The authors declare that the research was conducted in the absence of any commercial or financial relationships that could be construed as a potential conflict of interest. The reviewer AD and handling editor declared their shared affiliation at the time of the review.
