# Supplementary figures and images for: Single vs. Combined Therapeutic Approaches in Rats With Chronic Spinal Cord Injury
Source: Front Neurol. 2020 Mar 10;11:136. doi: 10.3389/fneur.2020.00136 (PMC7076126; doi:10.3389/fneur.2020.00136)

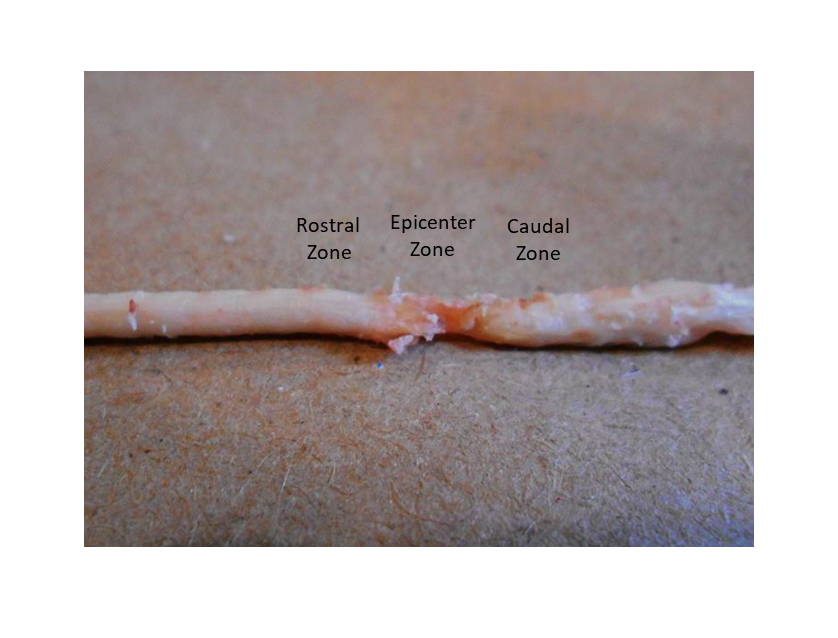

Supplement: Supplementary file 1 [file Image_1.TIF]

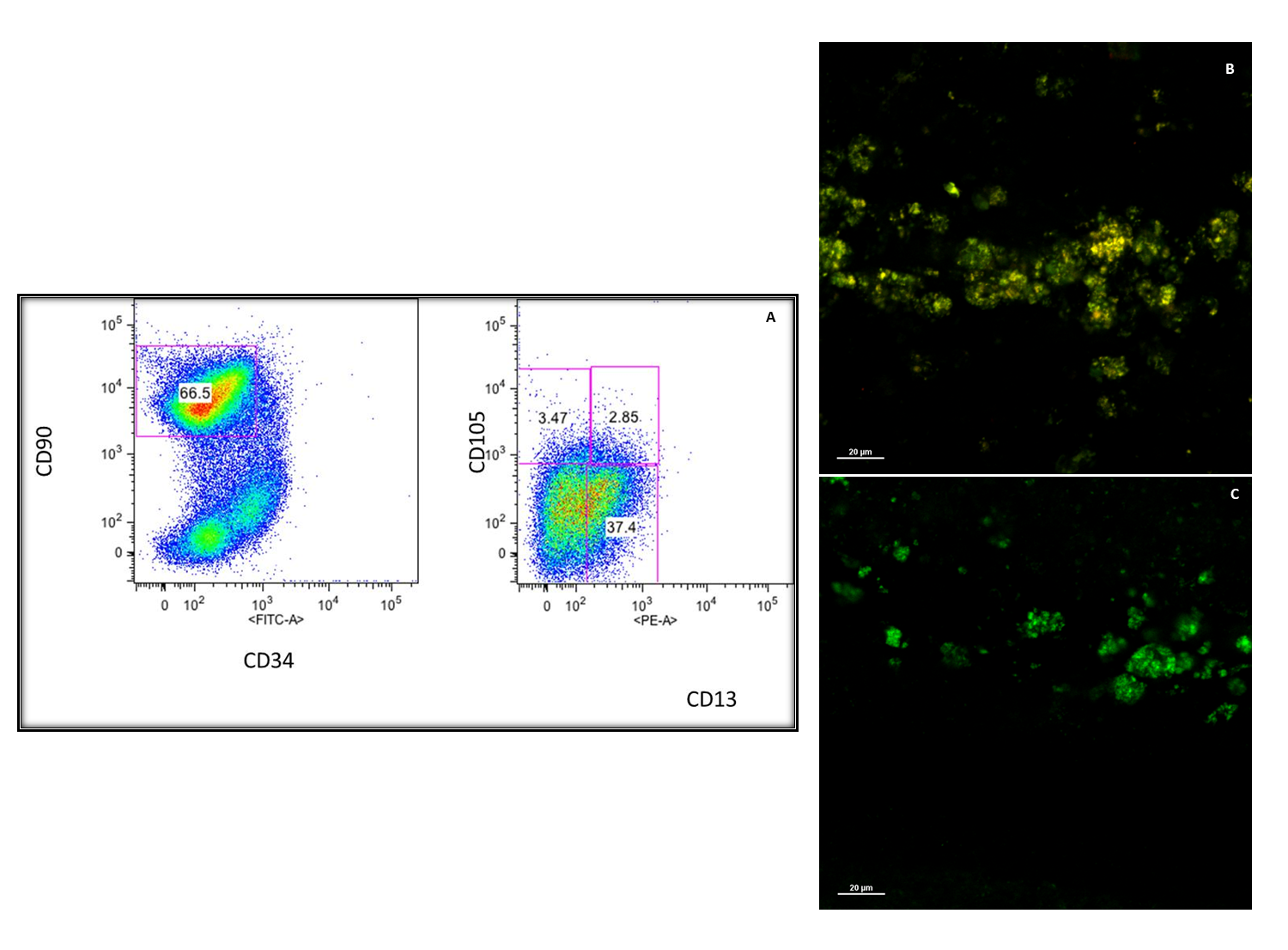

Supplement: Supplementary file 2 [file Image_2.TIF]

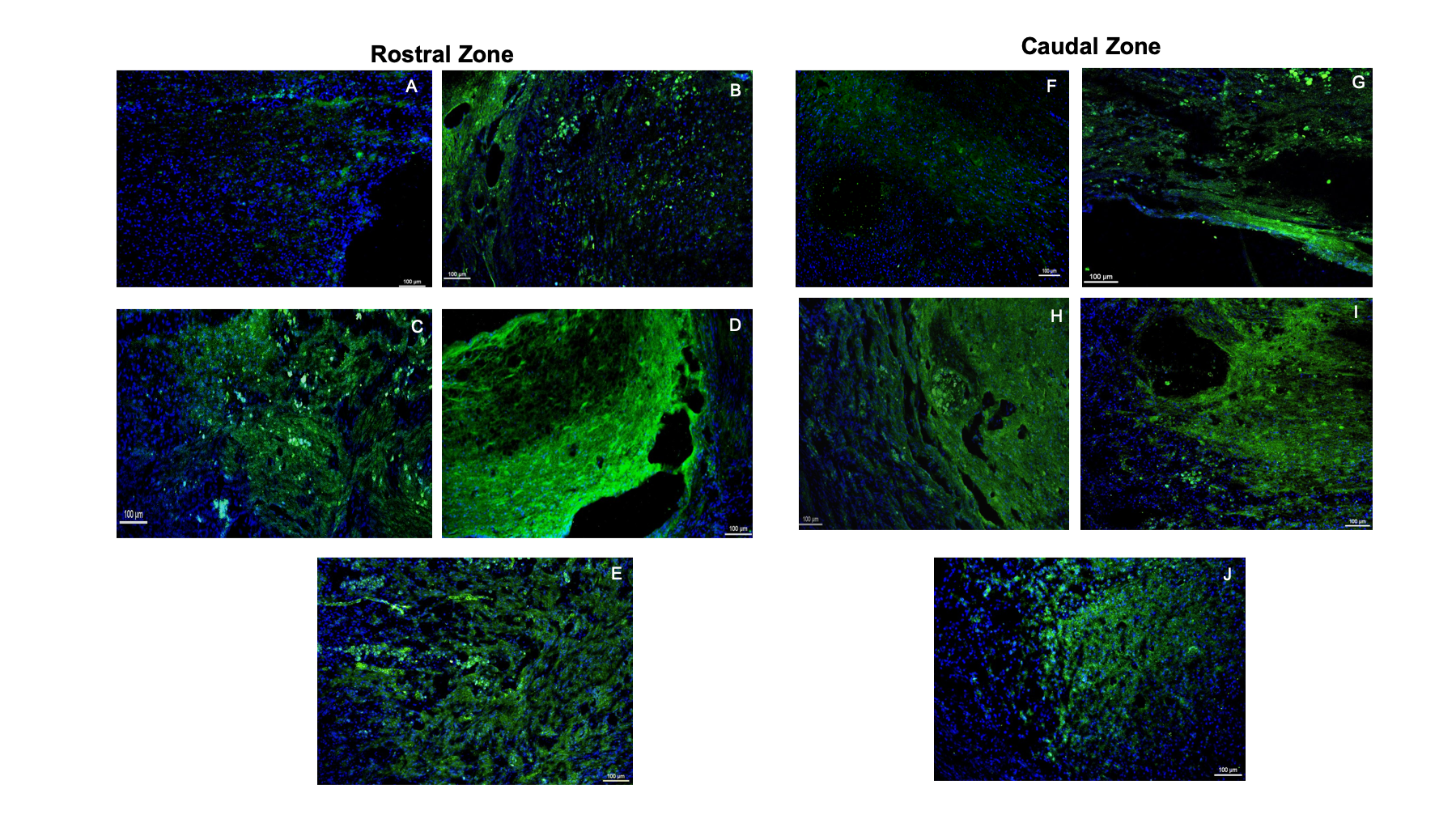

Supplement: Supplementary file 3 [file Image_3.TIF]

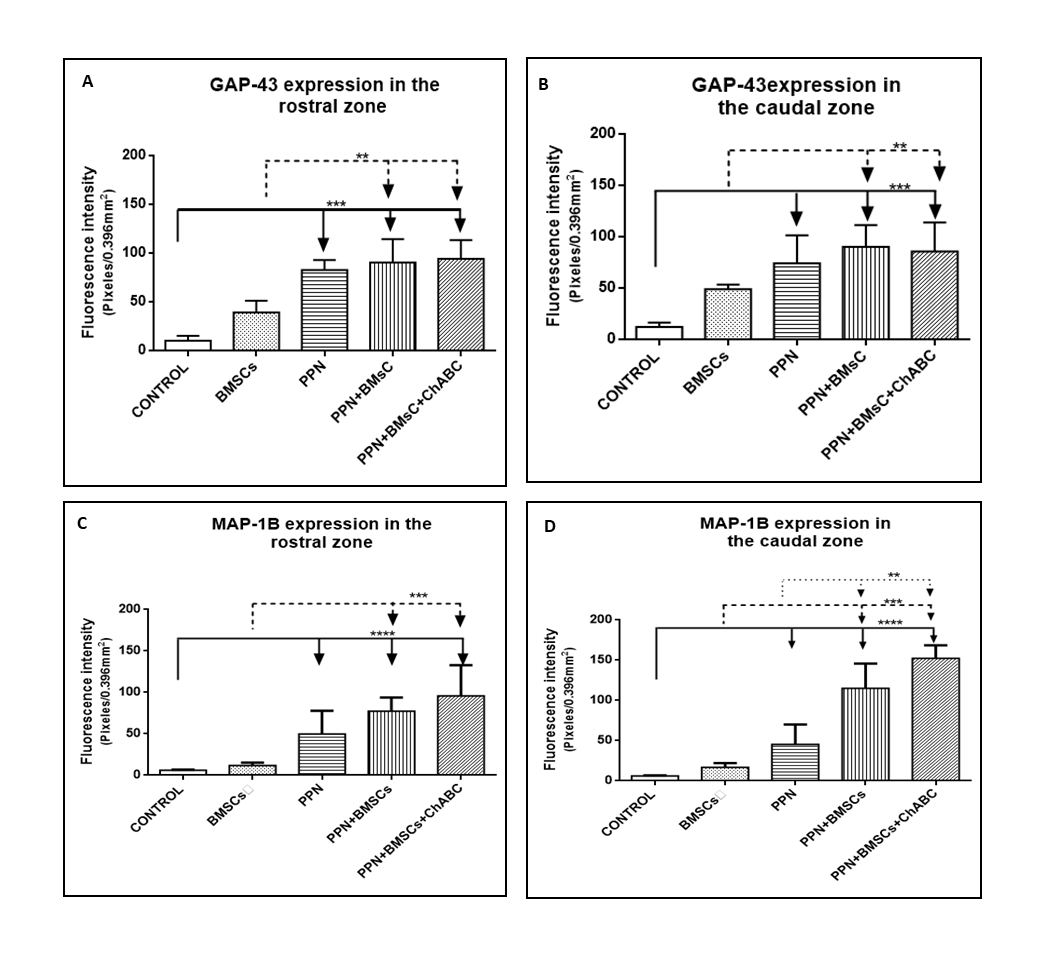

Supplement: Supplementary file 4 [file Image_4.TIF]

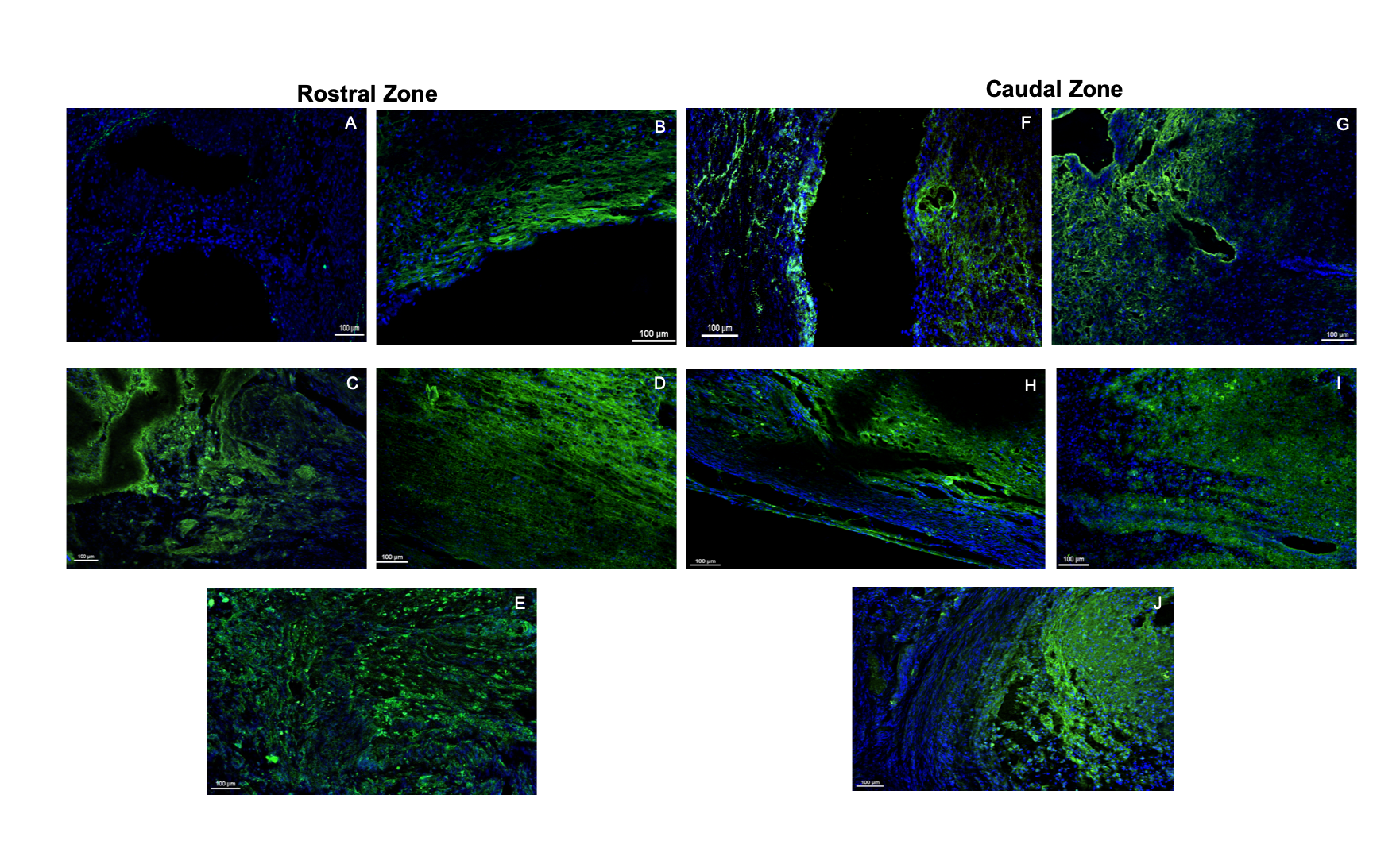

Supplement: Supplementary file 5 [file Image_5.TIF]

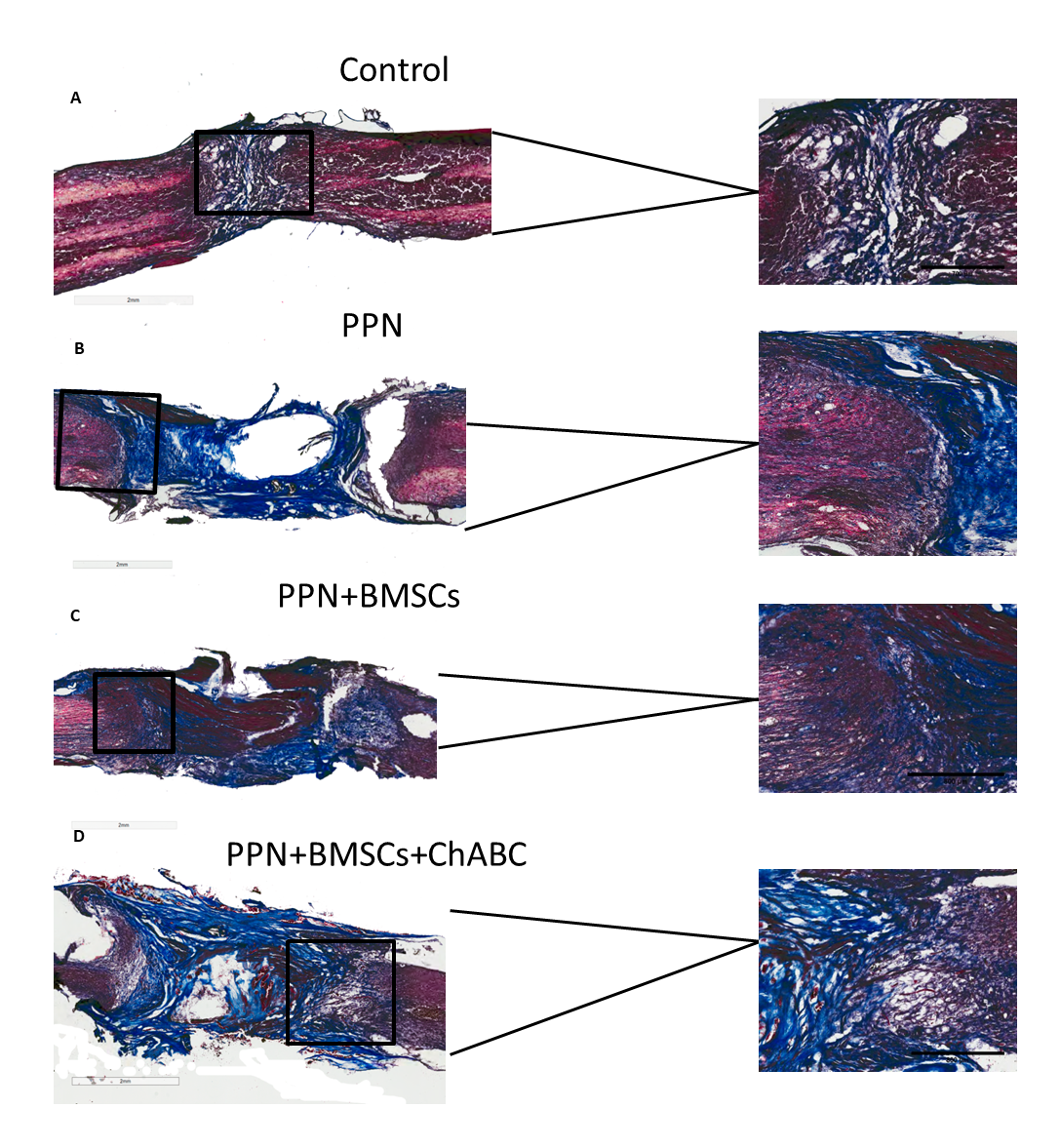

Supplement: Supplementary file 6 [file Image_6.TIF]

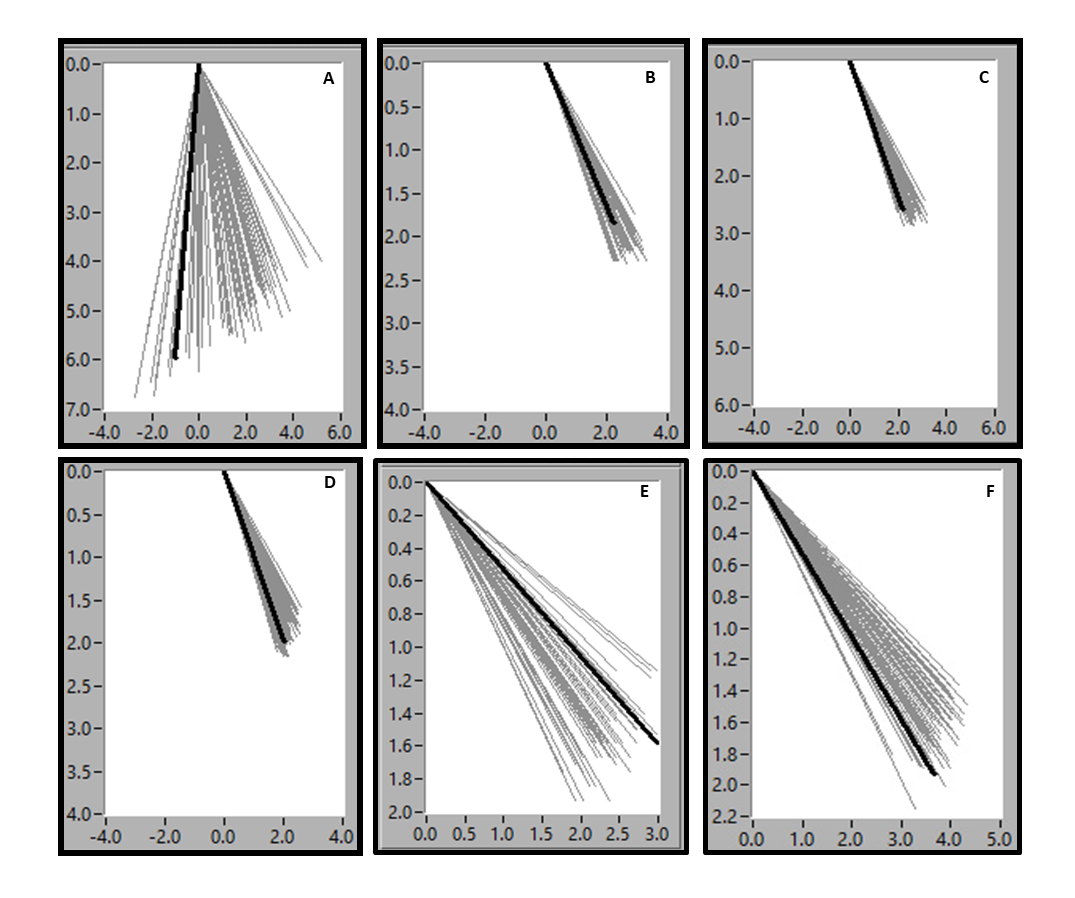

Supplement: Supplementary file 7 [file Image_7.TIF]
