# Supplementary material for: Single vs. Combined Therapeutic Approaches in Rats With Chronic Spinal Cord Injury
Source: Front Neurol. 2020 Mar 10;11:136. doi: 10.3389/fneur.2020.00136 (PMC7076126; doi:10.3389/fneur.2020.00136)
Supplement: Supplementary file 8 [file Data_Sheet_1.pdf]

SUPPLEMENTARY MATERIAL.

Characterization of the BMSC phenotype. The phenotype of cultured BMSCs was determined by flow cytometry. A sample of cells was centrifuged at 1500 rpm for five minutes and then incubated with the following primary antibodies: Cd105 (PE/CY7, anti-mouse Santa Cruz Biotechnology Inc., catalog no. 120409); Cd90.1 (PE, anti-rat Biolegend catalog no. 202523); Cd34 (ICO 115, FITC Santa Cruz Biotechnology Inc., catalog no. sc-7324), Cd13 (PE, Santa Cruz Biotechnology Inc., catalog no. sc-51522). All antibodies were used at a dilution of 1:200, in darkness, for 20 minutes, at 4°C. Cells were then washed twice with FACS Buffer before being centrifuged again at 1500 rpm for five minutes. The cells marked with Cd117 were incubated in darkness for two hours with the secondary antibody (Alexa 488 or 586, Molecular Probes Invitrogen 1:200). Afterward, cells were fixed in 4% paraformaldehyde for 1 hour and were finally quantified and analyzed with flow cytometry using the Cell Quest-Pro (BD Bioscience) program.

Morphological Changes. The histological appearance of the spinal cord after therapeutic approaches was determined by a trichrome Masson stain on two specimens of each group. Briefly, sections were hydrated and then placed in a Ferric Hematoxylin solution for 5 minutes. Thereafter, they were washed in distilled water and then placed in Biebrich scarlet for 5 minutes, washed in distilled water, and finally placed in analine blue for 5 minutes, washed in distilled water, dried, and covered with Entellan synthetic resin (Merck). Images were obtained with an Aperio CS2 microscope slide scanner (Leica Biosystem Germany).

Supplementary Figure 1. Macroscopic photography of an injured spinal cord. The rostral, epicentral, and caudal zones taken into account to perform histological section are shown.

Supplementary Figure 2. Characterization of BMSCs. As shown in panel A, 66.5% of cultured cells used for transplantation were positive for CD90, while 2.85% were double-positive for CD105/CD13, 37.4% were positive for Cd13, and 33% were positive for CD34, according to flow cytometry. Identification of BMSCs in spinal cord tissue shows immunoreactivity for CD90 (green) in panel B and for CD105 (green) in panel C.

Supplementary Figure 3. Representative images of GAP-43 expression at the spinal cord according to treatment. Photomicrographs in panels A-E correspond to the rostral zone; and F-J to the caudal zone. Immunoreactive fibers for GAP-43 (green). Nuclei dyed in blue, Hoechst. Scale bars: A-E = 100 μm; F-J = 50μm; K-O 100 μM.

Supplementary Figure 4. Quantification of GAP-43 and MAP-1B expression at the rostral and caudal zones: Plots in panels A and B show quantification of fluorescein density of GAP-43 at the rostral and caudal zones, respectively. Data are expressed as the mean ± S.D. (n = 6). Statistical analysis: Kruskal Wallis followed by U-Mann Witney. ***, $p<0.0001$; **, $p<0.05$. Plots in panels C and D show quantification of fluorescein density of MAP-1B at the rostral and caudal zones, respectively. Statistical analysis: Kruskal Wallis followed by U-Mann Withney. ***, $p<0.0001$; **, $p<0.05$ (difference between treatment groups U-Mann Withney). Data are expressed as the mean ± S.D (n = 6).

Supplementary Figure 5. Representative images of MAP-1B expression at the spinal cord according to treatment. Photomicrographs in panels A-E correspond to the rostral zone and F-J to the caudal zone. Immunoreactive fibers for MAP-1B (green). Nuclei dyed

in blue, Hoechst. Scale bars: A-E 100 μm; F-J 50μm; K-O 100 μm.

Supplementary Figure 6. Histological appearance of the spinal cord after therapeutic approaches. Representative sagittal spinal cord images. (A) Control specimen showing at the site of injury (rectangle). In specimens of rats that received single (B) or combined PPN transplantation: PPN+BMSCs (C) and PPN+BMSCs+ChABC (D), different degrees of host-graft apposition are shown in both rostral and caudal stumps. Masson's stain. Bar = 2 mm. Scale bar panel A-D 2 mm; enlargements 800 μm.

Supplementary Figure 7. Gait pendulum movement. In panel A, we observe the pendulum movement of an intact rat. In Panel B, we can see the pendulum movement of the control group. Panel C is the pendulum movement observed in the BMSC group. In panel D, we can observe the pendulum movement in the PPN group. In panel E, is the pendulum movement observed in the PPN+BMSCs group, and in Panel F, we can observe the pendulum movement in the PPN+BMSCs+ChABC group. All images were taken of the right leg.
